# Our Life and Times….Alan R. Gintzler, Ph.D. (1947–2021) Analgesics, Endogenous Opioids and the Variable of Sex

**DOI:** 10.3389/fpain.2021.753792

**Published:** 2021-09-07

**Authors:** Anne Z. Murphy, Mark Stewart, Martin Wessendorf

**Affiliations:** ^1^Neuroscience Institute, Georgia State University, Atlanta, GA, United States; ^2^School of Graduate Studies, State University of New York (SUNY) Downstate Health Sciences University, Brooklyn, NY, United States; ^3^Department of Neuroscience, University of Minnesota, Minneapolis, MN, United States

**Keywords:** obituary announcement, opioids, opiate tolerance, pain research, sex-related differences, life and times, spinal cord, steroids

On March 20, 2021, the scientific community lost a pioneer in the field of pain and opioid research: Dr. Alan Gintzler. Alan was born in Brooklyn, graduated *cum laude* with a degree in chemistry from Hunter College (1969) and a Ph.D. in Pharmacology from NYU Medical Center (1974). Following postdoctoral training with Dr. Sydney Spector at the Roche Institute for Molecular Biology (1974–1976), he joined the State University of New York (SUNY) Downstate Medical Center in 1980 in the Department of Biochemistry, becoming a full professor in 1990. He served as Chair of Biochemistry. Alan was recognized in 2017 as a SUNY Distinguished Professor in the Department of OB-GYN and served as a Board member of the Research Foundation of SUNY.

Alan's entire research career centered on opiates and their effects on pain, starting during the early 1970s, a period marked by the identification of opioid receptors and discovery of endogenous opioids. His work traveled two broad trajectories: first, biochemical mechanisms underlying opiate tolerance, and second, sex-related differences in pain and analgesia. This focus provided the foundation for a field of study dedicated to neuroendocrine and biochemical factors underlying sex differences in opiate action. His studies provided pivotal insights into cellular mechanisms of chronic pain, a devastating condition that disproportionately afflicts women. Alan's research focused on the molecular interface between ovarian steroids and endogenous-exogenous pain inhibitory mechanisms. His pioneering studies in the 1980s demonstrated that pregnancy-induced analgesia was due to endogenous spinal opioidergic circuits (1). These early studies, published in *Science* were the first to show that naturally occurring changes in gonadal steroid levels modulated activity of endogenous opioids. He identified a novel mechanism whereby opioids produced a differential degree of analgesia in males and females. Subsequent experiments showed that spinally mediated morphine analgesia in females, but not males, required co-activation of spinal mu and kappa-opioid receptors (MOR and KOR). Identification of female-specific MOR/KOR heterodimers established a novel pharmacological target for pain therapeutics in females.

Alan's studies of opiate tolerance suggested MORs could couple to Gs as well as Gi. The finding that MORs might be excitatory as well as inhibitory was significant, as it suggested that opioid tolerance could be the result of alternative signaling pathways that emerged in the presence of chronic opioids, as opposed to the previous assumption that tolerance was simply maintained *via* MOR uncoupling and/or downregulation. While this thinking was challenged, as opioids were seldom recognized to have excitatory effects. Today opioid agonist s mediated excitation is well-appreciated.

Alan's recent studies integrated the use of cell lines maintained in culture, *ex vivo* complex integrated neuronal preparations, and behaving animals to test the hypothesis that elevated Src activity and its associated downstream signaling cascades, were critical in development and maintenance of opioid tolerance. These recent studies epitomize Alan's continued dedication to translational research and his drive to facilitate development of safer and less addicting analgesics for chronic pain in males and females. Not surprisingly, with his research focus on sex and gender, and departmental affiliation with Ob-Gyn, Alan was a strong proponent of the need for focus on Women's health and was responsible for the oversight of an exciting NIH-funded training program focused on women's health—the Building Interdisciplinary Research Careers in Women's Health at SUNY.

In addition to his science, Alan was a remarkable mentor. He cared deeply about teaching and raising the next generation of scientists. He trained an impressive number of graduate students and postdoctoral fellows, many of whom remain in academia and continue to make significant contributions. Alan seldom wrote a common word when a more obscure one would do. That said, he published over 120 highly cited peer-reviewed manuscripts in premier journals, with his most recent study appearing in in February 2021 (2). Alan also maintained a high level of funding (primarily from NIDA) for over 40 years, a testament to his continued scientific imagination and efforts.

On a personal note, Alan was a devoted husband to his wife Ellen (see Figure 1). They met on a blind date in 1985, and they never looked back, being married for 32 years. His daughter, Ariella, was a constant source of pride and joy to him. Alan loved classical music and was a connoisseur of fine wine and food. He looked forward summers on the coast of Maine, where he and Ellen would kayak and hike. Alan was a man of ambition and energy, of quirks and kindness, and those of us who knew him, worked with him, or collaborated with him will miss him.

**Figure 1:**
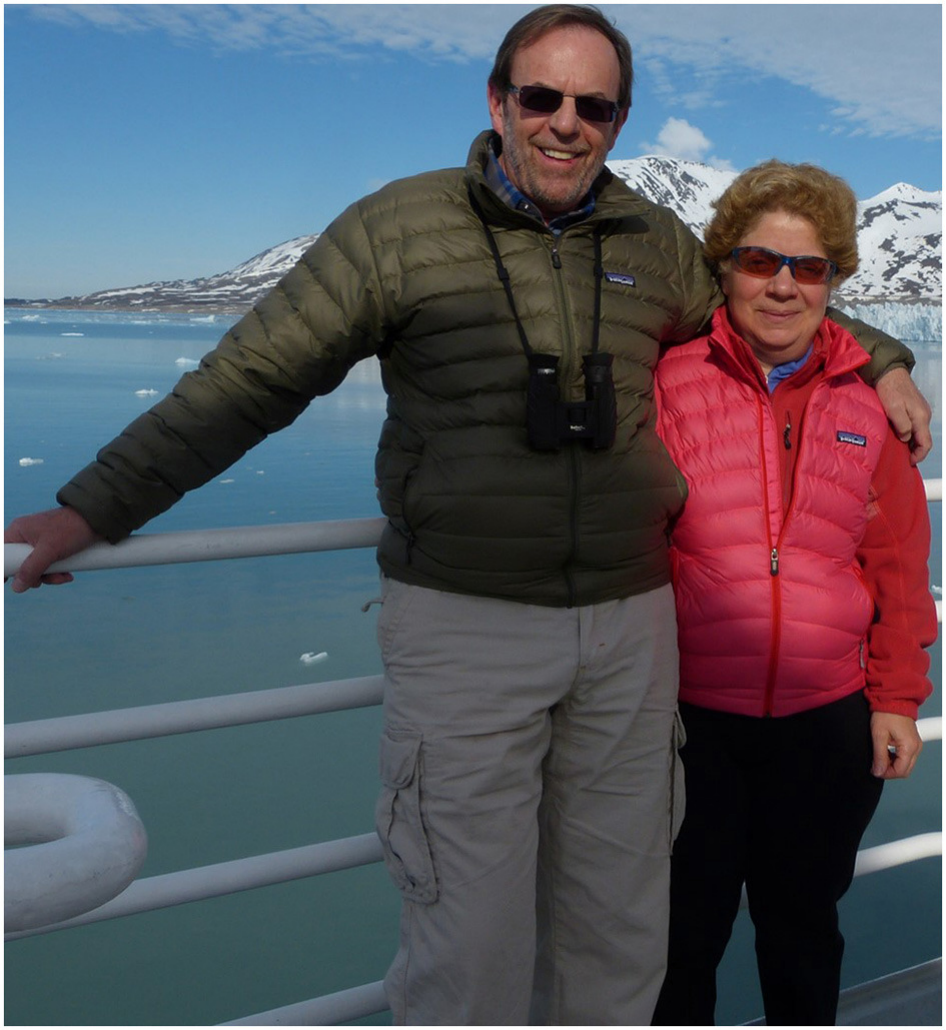
Alan and Ellen Gintzler on cruise.

## Author Contributions

All authors listed have made a substantial, direct and intellectual contribution to the work, and approved it for publication.

## Conflict of Interest

The authors declare that the research was conducted in the absence of any commercial or financial relationships that could be construed as a potential conflict of interest.

## Publisher's Note

All claims expressed in this article are solely those of the authors and do not necessarily represent those of their affiliated organizations, or those of the publisher, the editors and the reviewers. Any product that may be evaluated in this article, or claim that may be made by its manufacturer, is not guaranteed or endorsed by the publisher.
